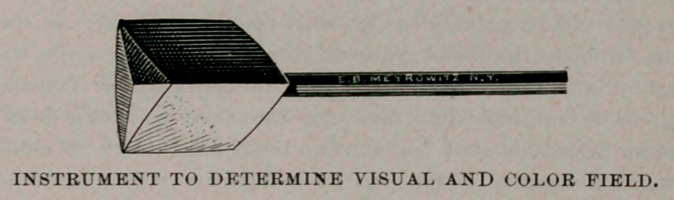# A Quick Test for the Visual and Color Fields

**Published:** 1898-02

**Authors:** Eugene N. S. Ringueberg

**Affiliations:** Lockport, N. Y.


### A QUICK TEST FOR THE VISUAL AND COLOR FIELDS.

BY EUGENE N. S. RINGUEBERG, M. D., Lockport, N. Y.

THE determination of the visual and color fields, while of great importance, is at times apt to be neglected on account of the time involved in their examination, even when made with a Claiborne rod with interchangeable balls or with different colored discs of paper. Furthermore, have found that there is apt to be quite a source of error due to the state of expectancy on the patient's part of seeing a certain color which he knows to be in the examiner's hand and will often call the color before he can accurately see it. Of course, we can occasionally change the color and so, to a certain degree, correct our observations, but this all takes more or less time and trouble.

To obviate these difficulties and to give the busy oculist an opportunity to determine approximately the visual and color fields

INSTRUMENT TO DETERMINE VISUAL AND COLOR FIELD.

as frequently as he may deem necessary, I have devised a little, simple instrument, which has been found very satisfactory for making a quick and approximately accurate test. This consists of a block of light wood, with three equal faces of 2 c.m. square, each ace being of a different color—red, blue and white respectively, care being taken to have the red and blue of nearly equal intensity. This block is mounted on a light rod. Its utility is at once apparent, as it will be seen that any of the colors can at once be brought forward by a very slight rotation of the rod between the thumb and finger holding it, without altering the position of the hand, or in any way giving the patient warning that a change has been made and a quite accurate idea of the size and shape of the color fields can be formed in a very few moments.

E. B. Meyrowitz, of New York, has undertaken to make the instrument, a cut of which is appended.

---

THE average man takes five and a half pounds of food and drink each day, amounting to one ton of solid and liquid nourishment annually. In seventy years he eats and drinks one thousand times his own weight. —*Ladies' Home Journal.*

## Figures and Tables

**Figure f1:**